# Role of CAP350 in Centriolar Tubule Stability and Centriole Assembly

**DOI:** 10.1371/journal.pone.0003855

**Published:** 2008-12-04

**Authors:** Mikael Le Clech

**Affiliations:** Department of Cell Biology, Max Planck Institute of Biochemistry, Martinsried, Germany; Institute for Research in Biomedicine, Spain

## Abstract

**Background:**

Centrioles are microtubule-based cylindrical structures composed of nine triplet tubules and are required for the formation of the centrosome, flagella and cilia. Despite theirs importance, centriole biogenesis is poorly understood. Centrosome duplication is initiated at the G1/S transition by the sequential recruitment of a set of conserved proteins under the control of the kinase Plk4. Subsequently, the procentriole is assembled by the polymerization of centriolar tubules via an unknown mechanism involving several tubulin paralogs.

**Methodology/Principal Findings:**

Here, we developed a cellular assay to study centrosome duplication and procentriole stability based on its sensitivity to the microtubule-depolymerizing drug nocodazole. By using RNA interference experiments, we show that the stability of growing procentrioles is regulated by the microtubule-stabilizing protein CAP350, independently of hSAS-6 and CPAP which initiate procentriole growth. Furthermore, our analysis reveals the critical role of centriolar tubule stability for an efficient procentriole growth.

**Conclusions/Significance:**

CAP350 belongs to a new class of proteins which associate and stabilize centriolar tubules to control centriole duplication.

## Introduction

Centrioles are required for the formation of the centrosome, flagella and cilia and are microtubule-based cylindrical structures that exhibit nine triplet tubules arranged around a nine-fold symetry carthweel structure [1]. The centrosome is the main microtubule organizing center in animal cells and is composed of a pair of centrioles surrounded by pericentriolar material. Despite its importance, the biogenesis of centriole is a poorly understood process. The centrosome duplication is initiated at the G1/S transition by the sequential recruitment of a set of conserved proteins under the control of Plk-4 and the related kinase Zyg-1 in *C.elegans* [2]–[5]. Using a centriole overduplication assay based on Plk-4 overexpression, we have previously proposed that in human cells hSAS-6, Cep135 and CPAP form a seed for the intiation of centriole growth [3]. Recently, in *C.elegans* a model for the elongation of centriolar tubules mediated by SAS-4 (homolog of CPAP) along a central tube formed by SAS-6 was proposed [6]. Subsequently, the procentriole is assembled by the polymerization of the first centriolar tubule named tubule A followed by the growth of the centriolar tubules B and C via an unknown mechanism involving several tubulin paralogs [7]. In spite of recent advances, the regulation of the centriolar tubule growth remains unknown. To monitor centrosome duplication in mammalian cells several assays based on the the formation of mutiple centrioles were developped. However, the centriole elongation process can not be analyzed with these assays. To this end we developped a new approach using synchronized RPE-1 cells and a microtubule-poisoning drug to reveal the role of CAP305 during centriolar tubule growth.

## Results

### Sensitivity of centriole growth to nocodazole

**Figure 1:**
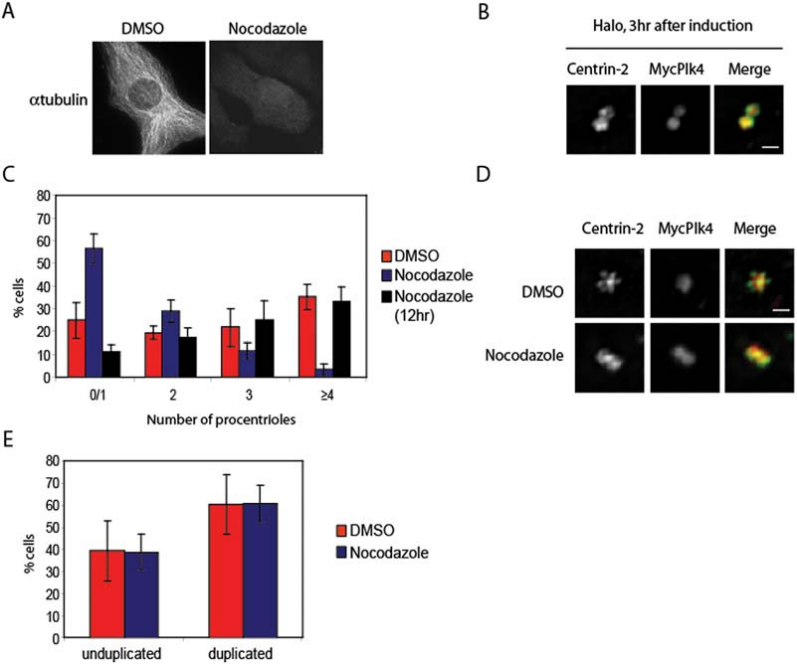
Nocodazole blocks centriole duplication at an early stage. (A) DMSO and nocodazole treated U2OS cells were stained with an antibody against α-tubulin. (B) Myc-Plk4 expression was induced for 3 hr in S-phase arrest U2OS cells and stained for Centrin (green) and Myc (9E10) (red) to illustrate a typical halo without new procentrioles. Note the presence of two parental centrioles. (C and D) Cells were treated with nocodazole (3.3 µM) simultaneously or 12 hr after the induction of Myc-Plk4. Control cells were treated with the vehicule DMSO (n = 3, ∼50 cells per condition). (C) Procentrioles were visualized using an anti-centrin staining and counted. The histogramm shows the number of procentrioles surrounding a parental centriole. Error bars represent SE. (D) The panel C shows the negative effect of nocodazole on procentriole assembly as visualized with centrin (green) and Myc (red) staining. Note that multiple procentrioles are arranged in a flower-like structure around the parental centriole in control cells. (E) U2OS cells were synchronized in mitosis with 25 µM noscapine and release for 3 hr before nocodazole (3.3 µM) treatment. The number of centriole was quantified using an anti-CP110 antibody 15 hr after nocodazole addition to the medium. Cells with unduplicated centrosomes exibit 2 CP110 dots staining 2 mother centrioles whereas cells with duplicated centrosomes exhibit 4 CP110 dots staining for 2 mother and 2 daughter centrioles. Error bars represent SE.

Centriole growth requires the addition of tubulin dimers or polymers to centriolar microtubules. The mechanism for the centriolar tubule polymerization is unknown but may share some similarities with microtubule growth. The effect of microtubule-poisoning drugs on centrosome duplication has not been tested in detail. It has been previously reported that colcemid treated cells have shorter daughter centrioles, although centriole initiation remains unaffected [8]. However, at a higher concentration, colcemid inhibits the initiation of centriole growth. More recently, centrosome overduplication in CHO cells has also been shown to be sensitive to nocodazole [9]. Alltogether, these results showed that depending on the concentration used, a microtubule-disrupting drug can either inhibits centriole elongation or block the initiation of centriole growth. To confirm and further detail the effect of a microtubule-poisoning drug on the centriole growth, we tested the effect of nocodazole on centrosome overduplication induced by Plk4 overexpression in S phase at a concentration that disrupts the microtubule network (Figure 1A). In order to have a sensitive read-out for centriole overduplication after Plk4 overexpression, we quantified the number of newly formed procentrioles per mother centriole. Indeed, the inducible expression of Plk4 in a U2OS/plk4 cell line results in the accumulation of Plk4 at the parental centriole which drives the formation of variable numbers of centrioles ranging from 2 to 9 as indicated by the staining of the centriolar marker centrin-2 [3](Figure S1A). The induction of Plk4 overexpression promotes the accumulation of centrosome proteins such as hSAS-6, CPAP, CP110 or Centrin-2 at the parental centriole forming a ring or a halo initiating the sprouting of procentrioles (Figures 1B and S1B). Consistent with previous work, application of nocodazole during the centriole overduplication decreased the proportion of cells with more than three procentrioles when compared to the control cells (Figure 1C). Concomitantly, the proportion of cells with no or one procentriole increased. Interestingly, mother centrioles without daughter centriole still recruited Plk4, and the formation of a halo as indicated by the accumulation of Centrin-2 was still apparent suggesting that while the initial events of the centriole duplication take place in the presence of nocodazole, procentriole growth may be defective (Figure 1D). The disruption of the microtubule network by nocodazole is unlikely to be responsible for this inhibition because the inactivation of the dynein mediated transport by a dominant negative approach has no effect on centrosome duplication [10]. Thus, these observations suggest that nocodazole may directly inhibit centriole overduplication by blocking the growth of centriolar tubules. Our previous work showed that the growth of procentrioles start between 6 and 16 hours after induction of Plk4 [3]. To determine whether nocodazole depolymerizes centriolar tubules, we added the drug 12 hours after the induction of Plk4 to allow for the initiation of centriolar tubule growth. Surprinsingly, drug addition at this stage had no effect on centriole overduplication indicating that the nocodazole did not depolymerize centriolar tubules (figure 1C). Together with the observation that nocodazole inhibits centriole duplication, our results indicate that nocodazole inhibits the polymerization of centriolar tubules early during the procentriole assembly process.

We next tested the effect of nocodazole during the normal one-round duplication of the centrosome in the same cell line. U2OS cells in G1 were treated with nocodazole and the duplication state of the centrosome were analysed 15 hours later by staining the centrosome with the centriole marker CP110 which is recruited at growing procentrioles [11]. The addition of the microtubule-disruptive drug did not inhibit the percentage of cells with four centrioles when compared to the control (Figure 1E). In agreement with previous work, our data showed that a microtubule-depolymerizing drug does not inhibit the initiation of the centriole growth and also reveals that the centriole overduplication process is more sensitive to nocodazole than the one-round centriole duplication. Given the different sensitivity to nocodazole in the two conditions, we propose that the centriole growth machinery during centriole overduplication is an exhausted system generating less stable procentrioles sensitive to microtubule-depolymerizing drugs. Furthermore, this result establishes that the polymerization of centriolar tubules is a robust process resistant to nocodazole and highlights the stability of growing centriolar tubule with respect to cytoplasmic microtubules. This raises the question of what controls procentriole stability during centrosome duplication.

### A cellular model for studying centrosome duplication

All mammalian cellular systems established to study centrosome duplication involve non-physiological stimulation of centrosome overduplication in transformed cell lines [3], [12]. Therefore, to answer the question how procentriole stability is regulated, a new cellular model needed to be developed to study the centrosome duplication in a non-transformed cell line under physiological conditions. The immortalized human cell line RPE-1 was choosen since it can easily be synchronized in G0 by 48 hr-serum starvation. In addition, the disruption of the microtubule network has no consequence on the cell cycle progression until M phase in these cells [13]. The duplication of the centrosome started in late G1 and proceeds during S, G2 and M phases [14]. The costaining of the centrosome with hSAS-6, a daugther centriole specific marker, and CP110 revealed that centriole duplication is initiated by the recruitment of hSAS-6 at both parental centrioles 15 hours after serum stimulation (Figure S2A and S2B). In S phase, the presence of three or four CP110 dots indicates that centriole elongation is in progress (Figure S2C).

### The microtubule binding protein CAP350 regulates procentriole stability

**Figure 2:**
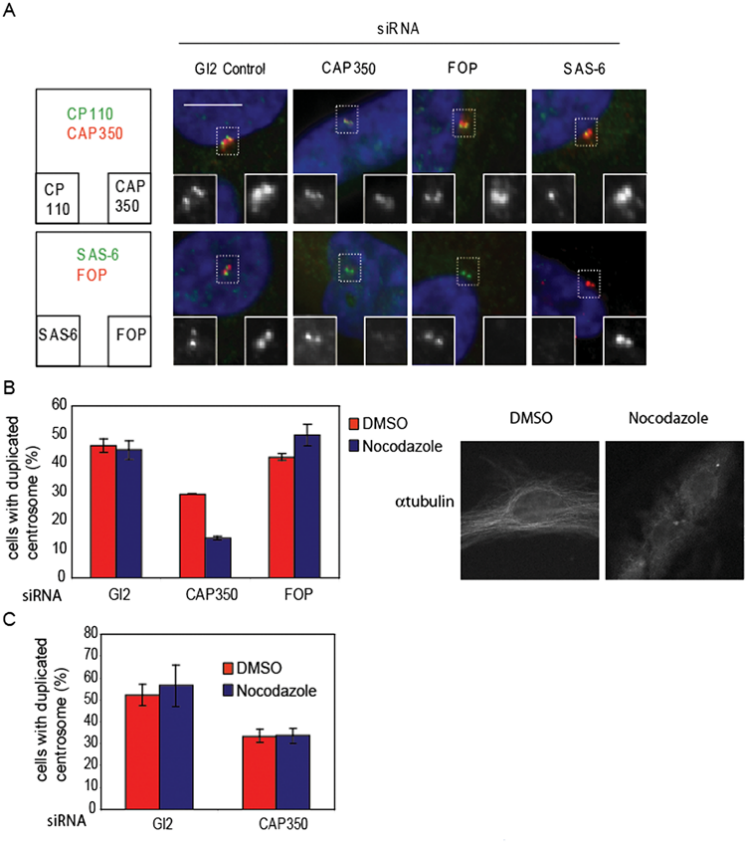
CAP350 protects centriolar tubules against the microtubule-depolymerizing activity of nocodazole. (A, B, C) RPE-1 cells were transfected for 24 hr with siRNA duplexes targeting CAP350, FOP, hSAS-6 or Gl2 for control. Then cells were starved for 48 hr before inducing the reentry into the cell cycle with a serum stimulation. Cells were processed for immunofluorescence microscopy 21 hr after serum addition, using the antibodies indicated and DAPI (blue). (B) siRNA transfected RPE-1 cells in G1 phase were treated with nocodazole (3.3 µM) or DMSO 12 hr after serum stimulation. The number of centriole was quantified using an anti-CP110 antibody 21 hr after serum addition. Cells with duplicated centrosomes exhibit 3 or 4 CP110 dots, (n = 3, ∼50 cells per condition). Error bars represent SE (right panel). DMSO and nocodazole treated RPE-1 cells were stained with an antibody against α-tubulin (left panel) (C) siRNA transfected RPE-1 cells in S/G2 phase were treated with nocodazole or DMSO 24 hr after serum stimulation for 1 hr. The number of centriole was quantified using an anti-CP110 antibody. Cells with duplicated centrosomes exhibit 3 or 4 CP110 dots, (n = 3, ∼50 cells per condition). Error bars represent SE.

Having established the cellular model, we then investigated the regulation of procentriole stability by testing the sensitivity of centriole biogenesis in RPE-1 cells to nocodazole after the depletion of potential factors. We focused our attention to CAP350 and FOP because they habor specific domains known to be involved in microtubule dynamics, a CAP-Gly domain and a LisH domain respectively. We have recently demonstrated that the centrosomal protein CAP350 recruits FOP to form a complex regulating microtubule anchoring [15]. Interestingly, CAP350 has been shown to stabilize microtubules via several microtubule binding domains suggesting that it could also stabilize centriolar tubules since CAP350 is associated with centrioles [16]. These features prompted us to investigate the role of this complex during centriole duplication initiation and elongation in RPE-1 cells by RNA interference experiments and nocodazole treatment. The duplication of the centrosome was monitored in S phase 21 hours after serum addition using an antibody targeting CP110. First, CAP350 and FOP depletion were assayed by immunofluorescence (Figure 2A). As previously reported [15], FOP depletion had no effect on CAP350 localization however CAP350 depletion delocalized FOP from the centrosome. As a control, we knocked down SAS-6 expression to prevent centrosome duplication and observed no effect on CAP350 and FOP localization (Figure 2A). Interestingly, the depletion of CAP350 and FOP had no consequence on the recruitment of SAS-6 at the centrosome indicating that they are not required for the initiation of the duplication of the centrosome (Figure 2A). As shown in the figure 2B, nocodazole had no effect on centrosome duplication in RPE-1 cells at a concentration disrupting cytoplasmic microtubules confirming that centriole duplication is resistant to nocodazole. Next, the effect of nocodazole on centriole duplication was compared after the knock down of CAP350 and FOP expression to the Gl2 control. We were unable to directly assess protein levels because CAP350 is not sufficiently abundant to be detected. Therefore, CAP350 and FOP depletion were checked by immunofluorescence. In the absence of nocodazole, CAP350 depletion had an effect on centrosome duplication while FOP had no effect. The addition of nocodazole strongly inhibited centriole duplication after CAP350 depletion but not after FOP depletion (Figure 2B). Thus, in contrast to FOP depletion, the knock down of CAP350 sensitized centriole duplication to nocodazole suggesting that CAP350 regulates procentriole stability independently of FOP. This result was validated by a second CAP350 specific siRNA (data not shown). To rule out a non-specific effect of the CAP350 depletion on the G1/S transition, levels of the late-S-phase-induced marker Cyclin B1 were measured by Western blot. The similar abundance of Cyclin B1 indicated that the cell cycle progress normaly in CAP350-depleted cells (Figure S3A). Additionaly, to investigate the specificity of nocodazole on centriole growth, we compared the proportion of cells positive for hSAS-6 with or without nocodazole as an indicator of the initiation of centriole duplication. The ratio of hSAS-6 positive cells between CAP350-depleted and Gl2 control cells remained unchanged after treatment demonstrating that when CAP350 protein levels are reduced, nocodazole inhibits specifically the growth of procentrioles but not the initiation of centriole duplication (Figure S3B). Note that the percentage of hSAS-6 positive cells is slightly lower in CAP350 depleted cells compared to the control, but as discussed below, a lower amount of hSAS-6 does not sensitize the centrosome duplication process to nocodazole.

As shown in the figure 1B, nocodazole interfered with procentriole growth at an early stage. Hence, the role of CAP350 later during procentriole elongation remains an open question. As mentioned before, the elongation of the centrioles proceeds during G2 and M phases. Therefore, we examined the stability of the growing centriole at the S/G2 transition which takes place between 24 and 30 hours after serum stimulation (data not shown). To this end, we treated the cells with nocodazole 24 hours after serum stimulation for 1 hour and quantified the number of duplicated centrosomes in control and CAP350 depleted cells. We found that nocodazole did not destabilize growing centrioles suggesting that CAP350 is not required for stabilizing growing centrioles in S/G2 (Figure 2C). Collectively, our data demonstrate that CAP350 has a centriolar tubule-stabilizing activity in growing procentrioles at an early stage of procentriole assembly.

### hSAS-6 and CPAP do not protect centriolar tubules against nocodazole

**Figure 3:**
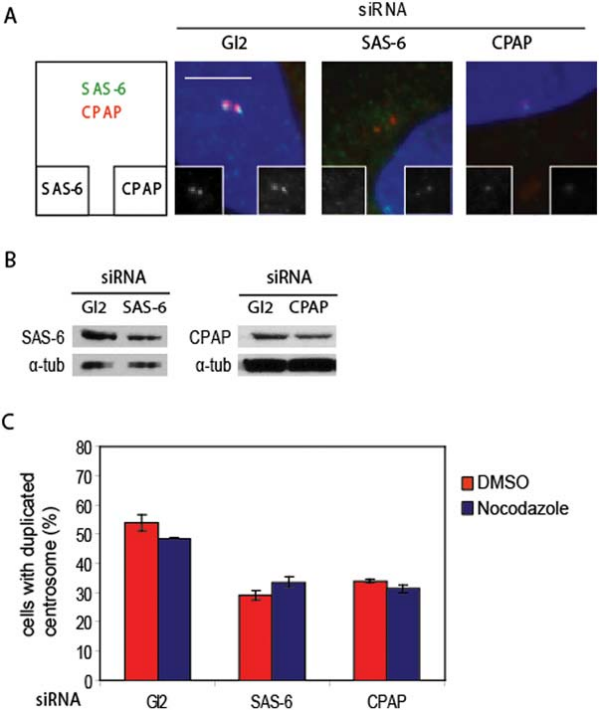
hSAS-6 and CPAP do not protect centriolar tubules against nocodazole. (A) RPE-1 cells were treated as described in the legend to Figure 2, using siRNA duplexes targeting hSAS-6, CPAP or Gl2 for control. Costaining were performed using anti-hSAS-6 (green), anti-CPAP (red) and DAPI (blue). (B and C) siRNA transfected RPE-1 cells in G1 phase were treated with nocodazole (3.3 µM) or DMSO 12 hr after serum stimulation before the recruitment of SAS-6. (B) Western blot on RPE-1 cells illustrating the partial depletion of hSAS-6 and CPAP by siRNA.(C) The number of centriole was quantified using an anti-CP110 antibody 21 hr after serum addition. Cells with duplicated centrosomes exhibit 3 or 4 CP110 dots, (n = 3, ∼50 cells per condition). Error bars represent SE.

Having established that CAP350 stabilizes procentrioles early in the assembly pathway of a new centrioles, we investigated whether the centriole duplication initiation process contribute to procentriole stability. Indeed, in the *C.elegans* model, hSAS-6 was proposed to form a tube allowing the SAS-4-mediated polymerization of the centriolar tubules along its lenght [6], [17]. Since the centrosome duplication machinery is conserved between species, hSAS-6 and CPAP, the human SAS-4 ortholog, could potentially interact with centriolar tubules to promote their growth and stabilization. In agreement with our previous finding [3], SAS-6 recruitment is dependent on CPAP as revealed by the lower abundance of hSAS-6 at the centrosome in CPAP depleted cells (Figure 3A). Additionally, we observed that the CPAP signal in hSAS-6 depleted cells is also slightly reduced showing that both proteins are interdependent. However, since CPAP is present at the centrosome before SAS-6 recruitment, a pool of CPAP is not dependent on hSAS-6 (data not shown). Therefore, this fraction of CPAP localizes probably to the pericentriolar region and when the centrosome duplication is initiated by hSAS-6, CPAP is recruited at the procentriole [18]. To address the role of these initiator proteins in the procentriole stability, we performed the procentriole stability assay described in the previous section after the partial depletion of hSAS-6 and CPAP by RNAi. Indeed a strong depletion of hSAS-6 or CPAP reduces severely centrosome duplication impeding the ability to analyse the centriole growth sensitivity to nocodazole (data not shown). Delivery of siRNA to cells decreased the level of SAS-6 and CPAP (Figure 3B). Confirming their role for the initiation of centriole growth, centrosome duplication was impaired following hSAS-6 and CPAP partial depletions. However in contrast to CAP350 depletion, the duplication of the centriole was still resistant to nocodazole treatment suggesting that they do not protect centriolar microtubules to nocodazole (Figure 3C). Hence, knowing that hSAS-6 and CPAP are thought to promote centriole elongation, our data indicate that hSAS6/CPAP do not stabilize the procentriole independently of their procentriole growth activity. In addition, we conclude that the increased sensitivity to nocodazole observed after CAP350 depletion is specific because defective initiation of centriole biogenesis did not sensitize the duplication process to this microtubule-depolymerizing drug.

### Procentriole stability is required for centriole growth

**Figure 4:**
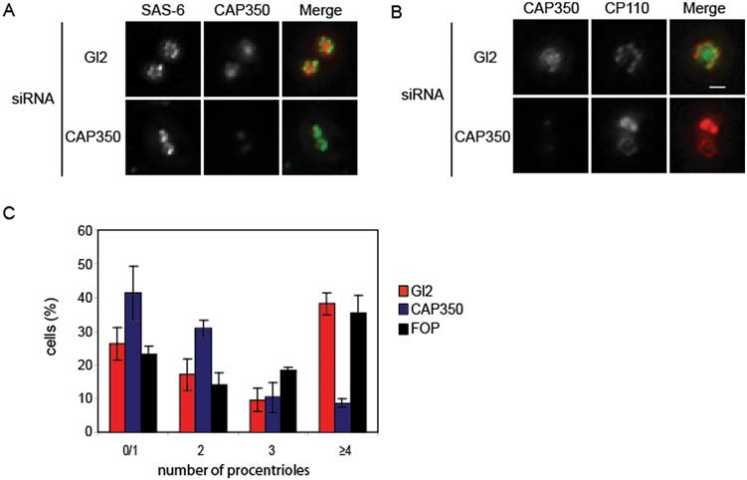
Stable centriolar tubules is required for the biogenesis of multiple procentrioles. (A and B) U2OS cells were transfected for 75 hr with siRNA duplexes targeting CAP350 and Gl2 for control. Then, Myc-Plk4 was induced for 21 hr in the continued presence of siRNA duplexes, and cells were processed for immunofluorescence microscopy using the antibodies indicated. (C) U2OS cells were transfected with siRNA duplexes targeting CAP350, FOP or Gl2 for control. Procentrioles were visualized using an anti-centrin staining and counted. The histogramm shows the number of procentrioles surrounding a parental centriole, (n = 3, ∼50 cells per condition). Error bars represent SE.

Our experiments demonstrate that CAP350 participates in a pathway stabilizing growing centriolar tubules. However, the depletion of CAP350 had no major effect on the duplication of the centrosome suggesting that either the stability of the procentriole is not essential for its growth or that some redundancy compensates the lack of CAP350. To address this question we investigated the consequence of CAP350 depletion on centriole overduplication in the Plk4-induced centriole overduplication assay. Since the centriole overproduction system generates less stable centrioles, we reasoned that the depletion of a protein involved in procentriole stability should reveal whether it is required for centriole growth or not. After Plk4 induction, CAP350 was associated with growing centrioles and the pericentriolar material as revealed by immunofluorescence (Figures 4A and 4B). As previously demonstrated, Plk4 induction promotes the accumulation of centrosomal proteins such as hSAS-6 and CPAP around the mother centriole [3]. These proteins form a ring or a halo structure promoting the growth of centrioles and the recruitment of CP110. In the absence of CAP350, both hSAS-6 and CP110 were localized around the mother centriole indicating that the initial events leading to centriole growth were not defective after CAP350 depletion in agreement with our observations in RPE-1 cells (Figure 4A and 4B). However, immunostaining indicated that CP110 positive structures differed between the control and CAP350 depleted cells. In CAP350 depleted cells, the CP110 staining formed a ring or a halo without distinctive dots suggesting that centriole growth is defective. Indeed, the growth of procentrioles enlarges the CP110 ring which at a critical size will form distinct CP110 dots at the distal tip of the newly formed procentrioles (Figure S1B). To validate this observation, we quantified the number of additional procentrioles produced after Plk4 overexpression in CAP350 and FOP depleted cells. As we were unable to directly assess CAP350 protein levels, protein depletion were checked by immunofluorescence. Whereas 42% of cells treated with the control siRNA had more than 3 procentrioles per mother centriole, only 10% of cells treated with the CAP350 siRNA showed efficient centriole overduplication (Figure 4C). As expected, the production of additional centrioles per mother centriole was not altered by FOP depletion. In this system, the depletion of CAP350 did not affect the percentage of SAS-6 or CPAP positive cells (data not shown). Taken together, these observations confirmed the requirement of CAP350 during centriole elongation and demonstrate that the centriole stability is required for centriole growth.

## Discussion

How centriole and basal bodies are assembled constitutes a long-standing unresolved question. Our findings provide evidence that centriole growth is regulated by the centriolar tubule-stabilizing activity of CAP350. The formation of a procentriolar “seed”, constituted notably by hSAS-6 and CPAP, promotes the assembly of a nascent procentiole under the control of Plk4 [3]. The stabilizing function of CAP350 is required early during the procentriole assembly process presumably when the first microtubule are polymerized. SAS-6 and CPAP control the polymerization of centriolar tubules independently of CAP350. Nonetheless, because hSAS-6 and CPAP are required for the initiation of centrosome duplication, their potential role in the stability of the procentriole cannot be totaly ruled out. Indeed, our assay cannot determine whether hSAS6 and/or CPAP have a potential coupled-microtubule stabilization/polymerization activity similar to some XMAP225/TOG family members. Therefore, we can only conclude that they do not function as a mere Microtubule Associated Protein like CAP350 (see below). Additional experiments are required to decipher the biochemical activity of hSAS-6 and CPAP. Finally, our data showed that the centriole overduplication system is less robust than the one-round centrosome duplication system which indicates that results obtained using centrosome overduplication systems should be analyzed with cautious.

A key question concerns the basis of the procentriolar stability mediated by CAP350. CAP350 localizes to the centriole and the pericentriolar material [15], [16]. Ultrastructural studies by electron microscopy were unsuccessful in revealing a specific signal for CAP350. However the CAP350 interactor FOP was successfully stained. FOP decorates centriolar tubule blades and knowing that CAP350 recruits FOP to the centrosome, we presumed that CAP350 interacts with centriolar blades (data not shown). By analogy to axon growth in neuron cells, CAP350 may function as a centriolar tubule-binding protein stabilizing the growing procentriole through a direct interaction via its multiple microtubule binding domains.

The highly stable nature of a centriole is conferred by the polyglutamylation of tubulins [19]. The polyglutamylation occurs during centriole elongation in G2/M phase when the tubulin polyglutamylase activity is high, making centrioles highly resistant to microtubule-depolymerizing drugs [3], [20]. However, we demonstrated that nocodazole does not depolymerize centriolar tubules induced by the overexpression of Plk4 in U2OS cells arrested in S phase suggesting that additional mechanisms regulate centriolar stability. This is consistent with the nocodazole resistance of the procentrioles in the absence of CAP350 in late S phase.

Microtubule depolymerization occurs by increased curvature of the end of protofilaments in the microtubule following GTP hydrolysis. Given the organization of the centriolar triplets, it is tempting to speculate that the close association of the centriolar tubules could prevent the microtubule peeling and protect them against a microtubule destabilizer such as nocodazole. The polymerization of tubules B and C requires ε-tubulin and δ-tubulin as revealed by the analysis of *Paramecium* and *Chlamydomonas* mutants [7], [21], [22]. In agreement with our hypothesis, these studies have suggested that the microtubule triplet function was to stabilize the centriole. Therefore, we propose the following model describing procentriole assembly. The formation of a “seed” constituted in part by hSAS6 and CPAP promote the initiation of the growth of the centriolar tubule A. At the onset of procentriole growth, microtubule stabilizing proteins such as CAP350 stabilizes the centriolar tubule A before the polymerization of the centriolar tubule B and C which probably offer additional interfaces for the interaction with other centriolar tubule binding proteins. This model provide an explanation of the early requirement of CAP350 and suggest that the assembly of the first centriolar tubule could be a critical step for the regulation of centriole duplication.

Our results demonstrate that unstable procentrioles fail to grow. This indicates that similarly to growing microtubule-based structures such as axons or cilia, centriolar tubules could grow and shrink under the control of external factors. Indeed, the recently reported centrosome inventory revealed several factors with potential centriolar-destabilizing activity [23]. Of particular interest are the microtubule severing enzymes such as Katanin or the microtubule-destabilizing Kinesins which are both known to regulate the flagellum length [24], [25].

Despite its potential importance for cancer progression, the question how structural anomalies appear in centrioles has not previously been adressed. In this study, we established the crucial role of stabilizing factors for normal procentriole assembly. The increased sensitivity of centriole overduplication to a microtubule-disrupting drug indicates that uncontrolled centrosome duplication may generate aberrant centriolar structures in cancer cells, either because of an altered centriolar tubule stabilizing pathway or by an increased expression of centriolar destabilizing factors. Consistent with this hypothesis, application of high concentrations of vinblastine generates daughter centrioles with aberrant structures reminiscent of what is observed in cancer cells [26]–[28]. A better understanding of the regulation of procentriole stability may shed some light on the role of centrosome abnormalities during cancer progression.

## Materials and Methods

### Antibodies

Rabbit polyclonal antibody was raised at Charles Rivers laboratories (Elevages Scientifique des Dombes, Charles River laboratories, Romans, France) against GST-CP110 (aa 1–149) and then purified according to standard purification. Rabbit anti-CAP350, anti-FOP, anti-CPAP and anti-centrin-2 were previously described [3] and antibody against α-tubulin and acetyl-tubulin were purchased from Sigma (Taufkirchen, Germany). The monoclonal antibodies against hSAS-6 and CPAP were previously described [3].

### Cell Culture and transfection

U2OS and the U2OS/plk4 cell line were cultured as described previously [3]. hTERT-RPE1 cells were grown in DME nutrient mixture, Ham's F12 (Sigma-Aldrich) supplemented with 10% FCS, penicillin-streptomycin, 2 mM glutamine, and 0.348% sodium bicarbonate. siRNA transfections were performed using Oligofectamin (Life Technologies, Karlsruhe, Germany).

### siRNA experiments and procentriole stability assay

CAP350, FOP, CPAP and hSAS-6 were depleted using siRNA duplex oligonucleotides (Qiagen and Dharmacon) targeting the sequences described previously [3], [15]. A duplex targeting luciferase (GL2) was used for control [3]. RNA oligonucleotides were used at 200 nM, except for the partial depletion of hSAS-6 and CPAP where 100 nM of siRNA were used. hTERT-RPE1 cells (provided by L. Kohen, Universiätsklinikum Leipzig, Leipzig, Germany) were grown on acid-treated, sterilized glass coverslips and transfected for 24 h with different siRNA duplexes. Go state was induced in confluent cells by continued culturing in serum-free medium for another 48 h. Cell cycle reentry was induced by 10% FCS addition. 12 hr after serum stimulation, cells were incubated for 9 hr with 3.3 µM nocodazole.

### Microscopic techniques

Cells were prepared for immunofluorescence as previously described [3]. RPE-1 cells were analysed using a microscope (Axioskop; Carl Zeiss Microimaging, Inc.) equipped with a 63× NA 1.4 plan apochromat oil-immersion objective and standard filter sets, a 1,300×1,300 pixel cooled charge-coupled device camera (CCD-1300-Y; Princeton Instruments), and Metavue software (visitron Systems). Alternatively, centriole overduplication in the U2OS/Plk4 cell line were analysed using a Deltavision microscope on a Nikon TE200 base (Applied Precision, Issaquah, WA) equipped with a APOPLAN ×100 NA 1.4 plan oil-immersion objective. Serial optical sections obtained 0.3 µm apart along the Z-axis were processed using Softworx (Applied Precision).

## Supporting Information

Figure S1 Plk4-induced centriole biogenesis (A and B) U2OS cells were treated as indicated in the legend Figure 1. (A) Centrosome was stained with anti-centrin (green) to visualize centrioles and anti-hSAS6 (red) which accumulates around the mother centriole. (B) Myc-Plk4 expression was induced for 3 hr and 24 hr. Centrosome was stained with Myc 9E10 (green) and anti-CP110 (red). At 3 hr, no flower-like structure is observed hence, CP110 is accumulated around the MycPlk4 signal forming an outer ring. At 24 hr, the CP110 staining is organized like a flower-like structure revealing that procentrioles are growing.
(0.94 MB TIF)

Figure S2 Description of centrosome duplication in RPE-1 cells using centriolar markers. (A and B) RPE-1 were synchronized in G0 by serum starvation and then restimulated with 10% serum. Centrioles were stained with an anti-hSAS-6 (green) and anti-CP110 (red) and DNA was stained with DAPI (blue) at different time points. (A) the panel A shows all hSAS-6 and CP110 staining patterns observed. Note that cells with 3 or 4 CP110 dots harbor separated centrosomes for an easier visualisation of the centrioles. (B) The different hSAS-6 and CP110 staining patterns were quantified for each indicated time points (100 cells counted at each time points). (C) Centrioles was stained with anti-acetyl-tubulin (green) to visualize the cilium and with an anti-CP110 (red). DNA was stained with DAPI. Note that separated centrosomes indicate that the cells are in G2 and that cells with three CP110 dots still exibit a cilium preventing the recruitment of CP110.
(1.16 MB TIF)

Figure S3 Nocodazole inhibits specificaly centriolar tubule growth. RPE-1 cells were treated as indicated in the legend figure 2. (A) Total cell lysates from CAP350-depleted or control cells were collected 21 hours after serum stimulation and probed using the antibodies indicated. Cep135 levels provide a loading control. (B) SAS-6 positive cells were quantified in Gl2 and CAP350-depleted cells. The histogramm shows the ratio CAP350/Gl2 in control and nocodazole treated cells. In control and nocodazole treated cells, the ratio is <1 due to a lower abundance of SAS-6 in CAP350, (n = 3, ∼50 cells per condition). Error bars represent SE.
(0.81 MB TIF)
