# Supplementary figures and images for: Role of CAP350 in Centriolar Tubule Stability and Centriole Assembly
Source: PLoS One. 2008 Dec 4;3(12):e3855. doi: 10.1371/journal.pone.0003855 (PMC2586089; doi:10.1371/journal.pone.0003855)

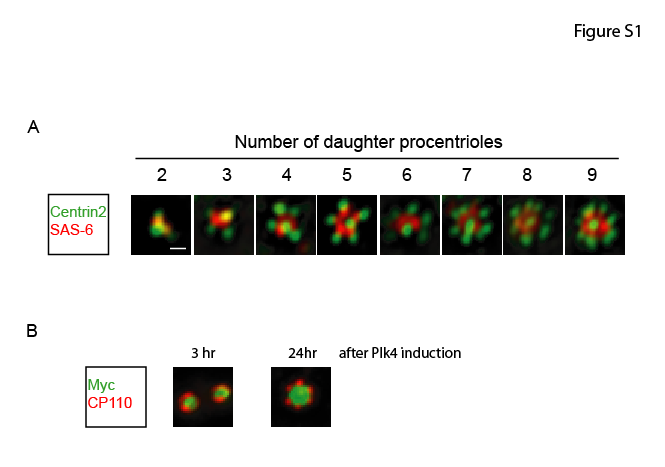

Supplement: Figure S1 — Plk4-induced centriole biogenesis (A and B) U2OS cells were treated as indicated in the legend Figure 1. (A) Centrosome was stained with anti-centrin (green) to visualize centrioles and anti-hSAS6 (red) which accumulates around the mother centriole. (B) Myc-Plk4 expression was induced for 3 hr and 24 hr. Centrosome was stained with Myc 9E10 (green) and anti-CP110 (red). At 3 hr, no flower-like structure is observed hence, CP110 is accumulated around the MycPlk4 signal forming an outer ring. At 24 hr, the CP110 staining is organized like a flower-like structure revealing that procentrioles are growing. (0.94 MB TIF) [file pone.0003855.s001.tif]

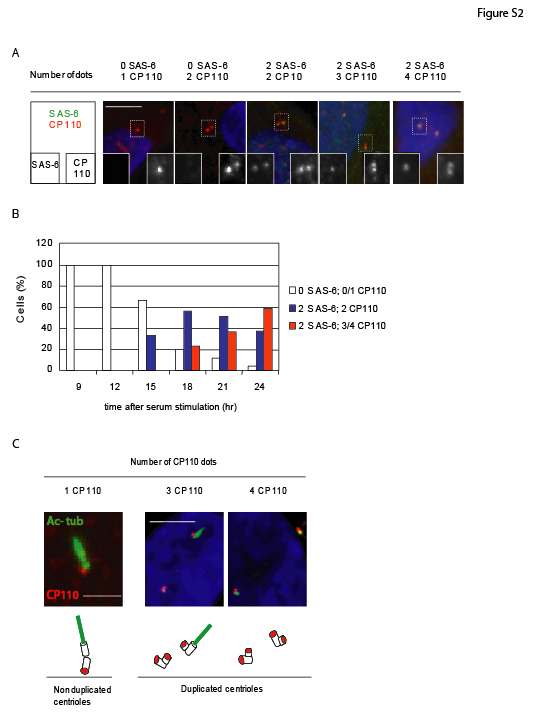

Supplement: Figure S2 — Description of centrosome duplication in RPE-1 cells using centriolar markers. (A and B) RPE-1 were synchronized in G0 by serum starvation and then restimulated with 10% serum. Centrioles were stained with an anti-hSAS-6 (green) and anti-CP110 (red) and DNA was stained with DAPI (blue) at different time points. (A) the panel A shows all hSAS-6 and CP110 staining patterns observed. Note that cells with 3 or 4 CP110 dots harbor separated centrosomes for an easier visualisation of the centrioles. (B) The different hSAS-6 and CP110 staining patterns were quantified for each indicated time points (100 cells counted at each time points). (C) Centrioles was stained with anti-acetyl-tubulin (green) to visualize the cilium and with an anti-CP110 (red). DNA was stained with DAPI. Note that separated centrosomes indicate that the cells are in G2 and that cells with three CP110 dots still exibit a cilium preventing the recruitment of CP110. (1.16 MB TIF) [file pone.0003855.s002.tif]

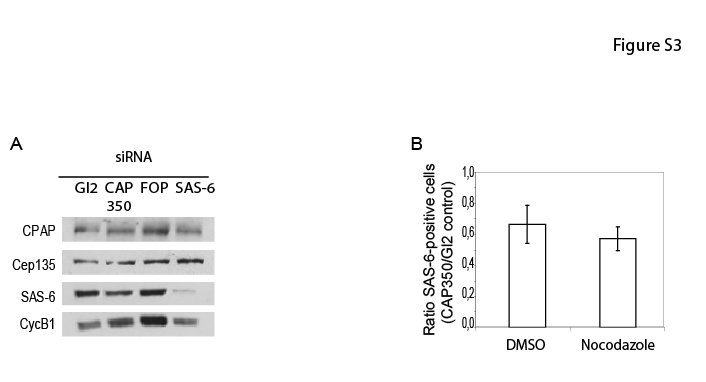

Supplement: Figure S3 — Nocodazole inhibits specificaly centriolar tubule growth. RPE-1 cells were treated as indicated in the legend figure 2. (A) Total cell lysates from CAP350-depleted or control cells were collected 21 hours after serum stimulation and probed using the antibodies indicated. Cep135 levels provide a loading control. (B) SAS-6 positive cells were quantified in Gl2 and CAP350-depleted cells. The histogramm shows the ratio CAP350/Gl2 in control and nocodazole treated cells. In control and nocodazole treated cells, the ratio is <1 due to a lower abundance of SAS-6 in CAP350, (n = 3, ∼50 cells per condition). Error bars represent SE. (0.81 MB TIF) [file pone.0003855.s003.tif]
